# Supplementary material for: Gene-Expression Programs in Salivary Gland Adenoid Cystic Carcinoma Analyzed Using Single-Cell and Spatial Transcriptomics
Source: bioRxiv. 2025 Sep 5:2025.09.01.673548. Preprint. [Version 1] doi: 10.1101/2025.09.01.673548 (PMC12424767; doi:10.1101/2025.09.01.673548)
Supplement: Supplement 1 [file media-1.pdf]

Integrative Multi-Omics Reveals Gene-Expression Programs in Salivary Gland Adenoid Cystic Carcinoma through Single-Cell and Spatial Transcriptomics

Ifeoma Ebinumoliseh, Gopikrishnan Bijukumar, Kendall Hoff, Kathryn Brayer, Elaine Bearer, Scott Ness, Jeremy Edwards

SUPPORTING INFORMATION:

1A

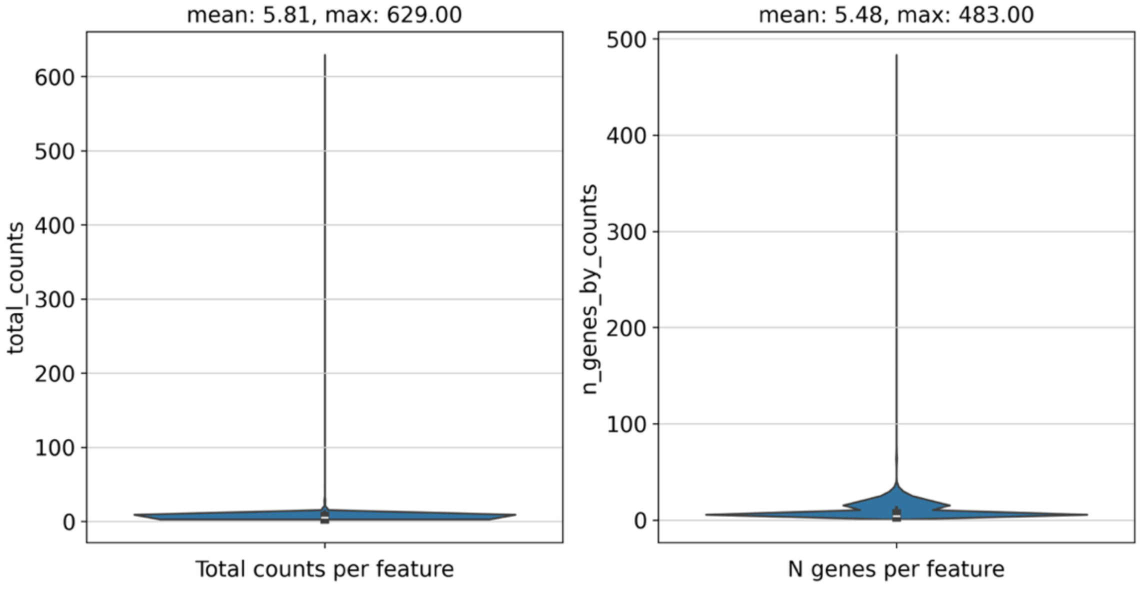

1B

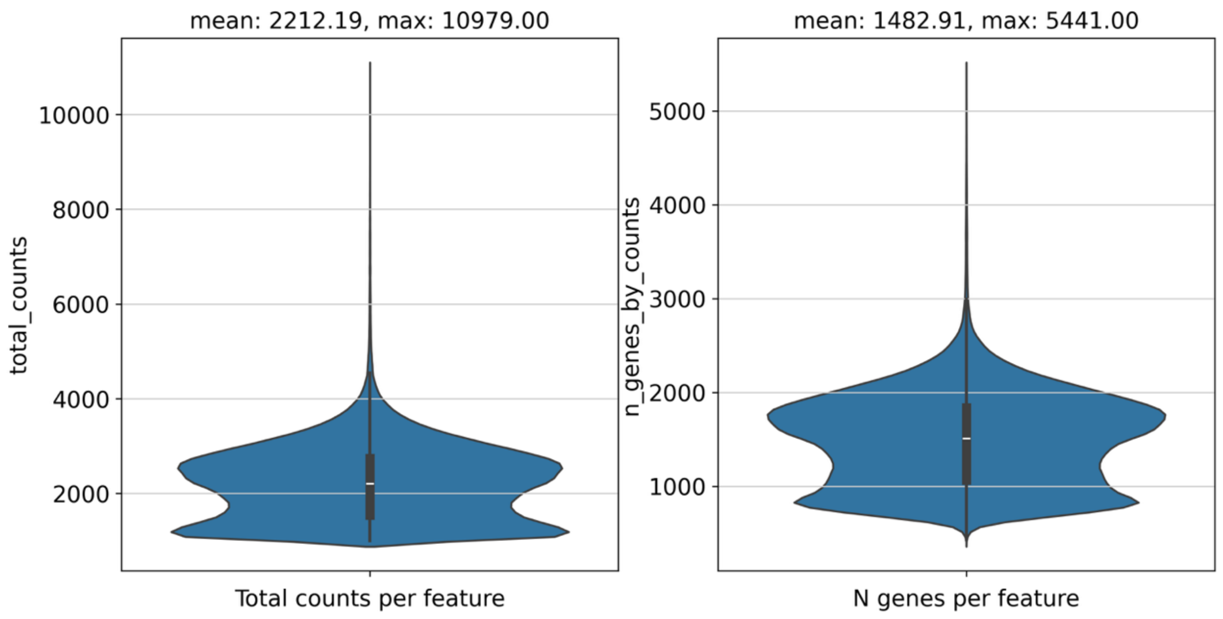

Figure. S1A and B. Preprocessing Quality control metrics of the spatial transcriptomics query dataset. (A) Total counts per feature and N-genes per feature before binning. (B) Total counts per feature and N-genes per feature after binning

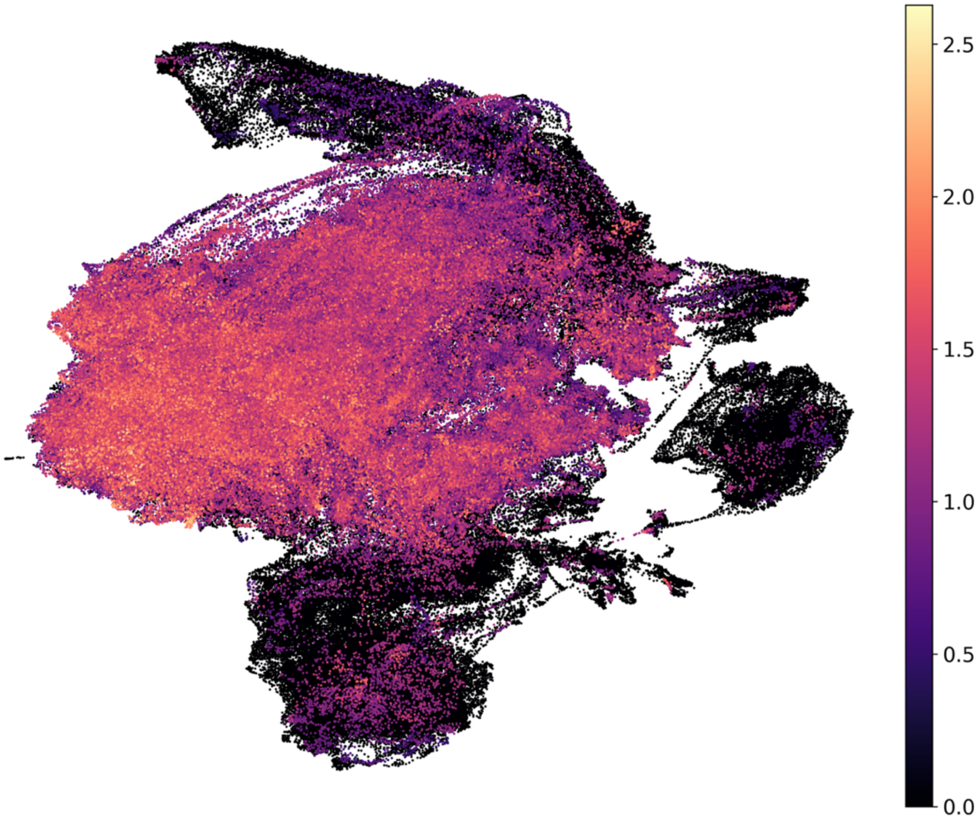

Figure S2. MYB Expression in UMAP of Spatial Data

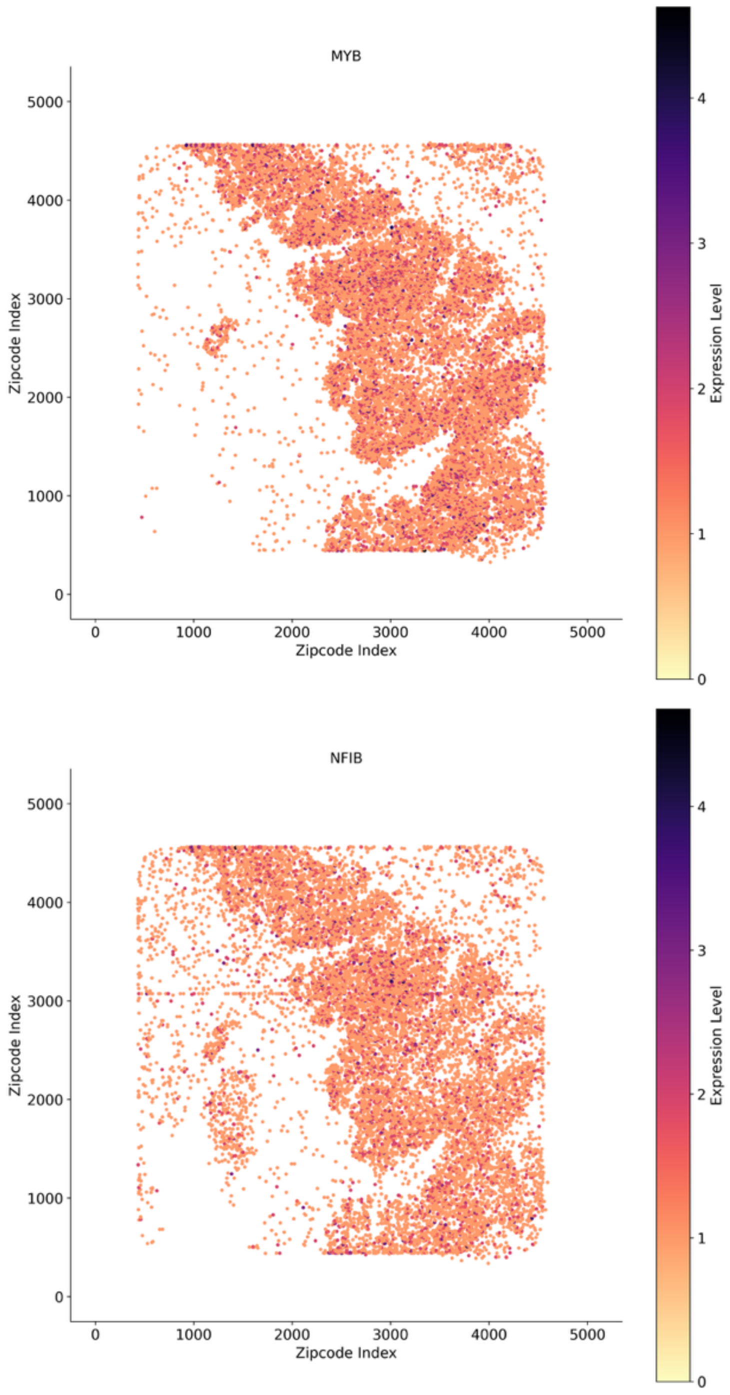

Figure S3. Mapped Zip Codes Showing MYB and NFIB Expression in Tissue Architecture

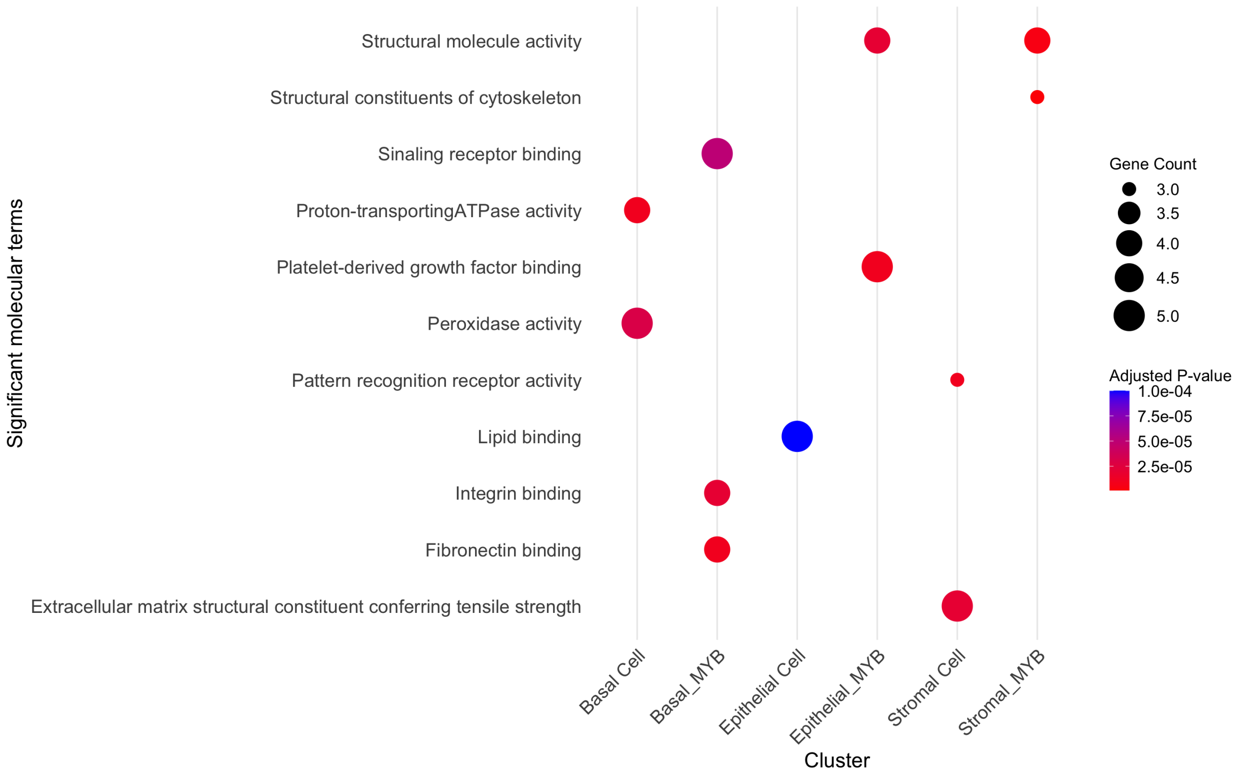

Figure S4. Pathway Enrichment of MYB and Non-MYB Expressing Stromal, Epithelial and Basal Clusters - Significant molecular terms

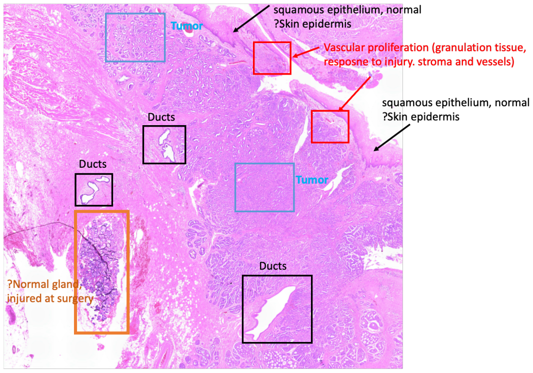

Figure S5. Pathology Annotation of Tissue

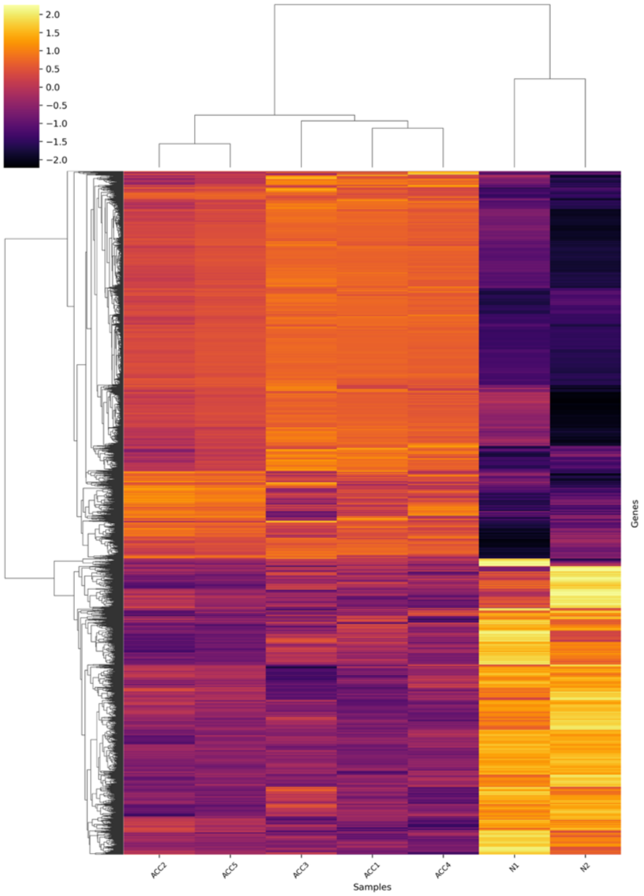

Figure S6:Heatmap of top 8,000 differentially expressed genes of normal control (N1 and N2) vs SGACC (ACC1 -ACC5) samples (n = 7)

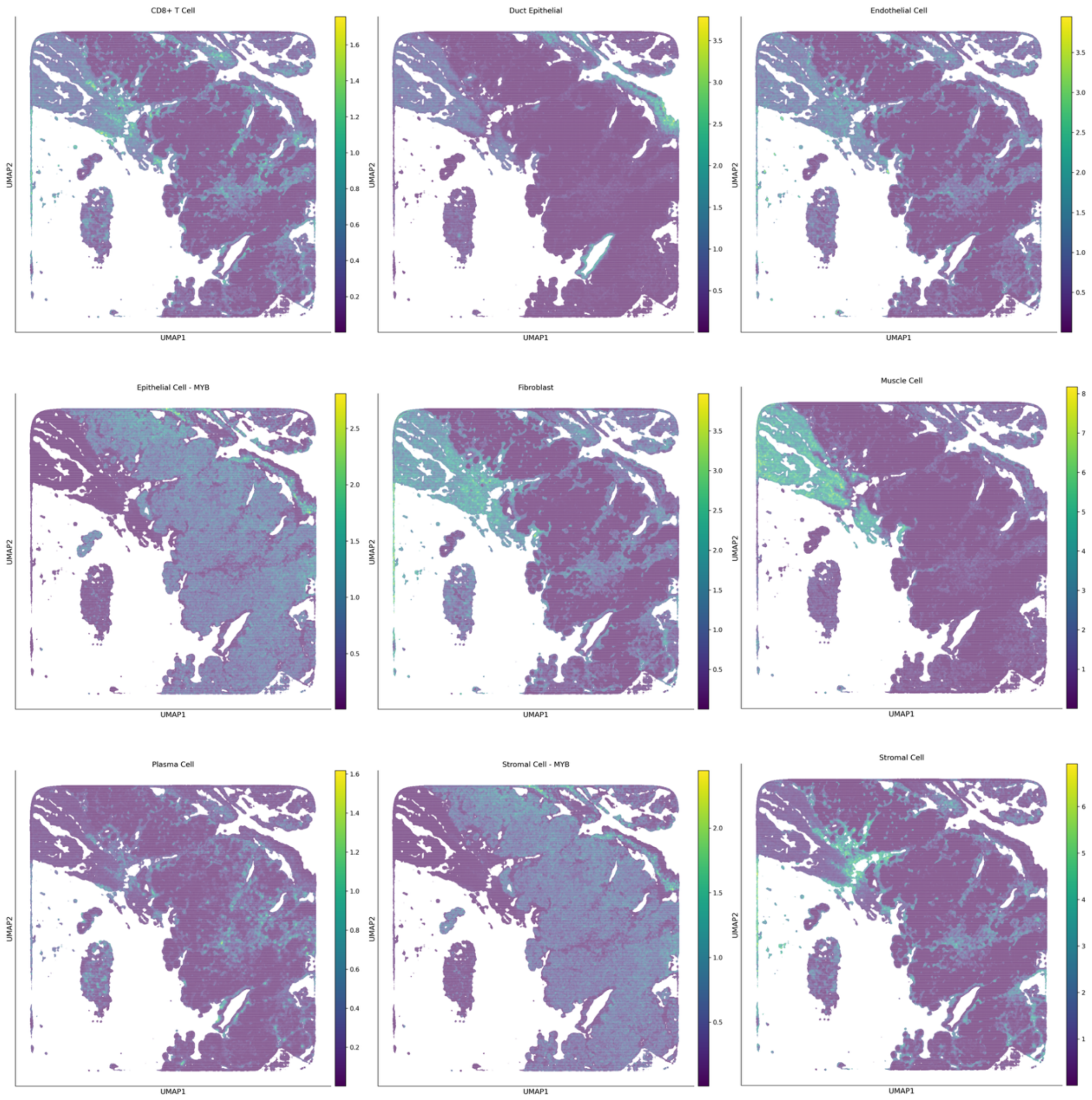

Figure S7. Spatial Localization of Individual Cells Within the Tissue Architecture

Table S1:

Marker genes for each cell types

Epithelial Cell-MYB	Basal Cell-MYB	CD8+ T cell	Duct Epithelial Cell	Endothelial Cell	Epithelial Cell	Fibroblast Cell	Muscle Cell	Plasma Cell	Stromal Cell	Stromal Cell-MYB
**AZGP1**	KRT5	GZMH	MUC5B	MGP	KRT13	COL1A1	TNNT3	IGKC	CLEC3B	ALDH1A3
**ITGB4**	KRT17	KLRG1	PIGR	EGR3	SPRR2A	COL3A1	CKM	IGHA2	COL1A1	EGR3
			BPIFB2		SBSN	COL1A2	MB	IGLC2	HBA2	NR4A1
					LY6D		TTN	JCHAIN		
							STAC3			
